# (*E*)-3-Anilino-2-benzoyl-3-(methyl­sulfan­yl)acrylonitrile

**DOI:** 10.1107/S1600536812013475

**Published:** 2012-03-31

**Authors:** Hatem A. Abdel-Aziz, Hazem A. Ghabbour, Suchada Chantrapromma, Hoong-Kun Fun

**Affiliations:** a Department of Pharmaceutical Chemistry, College of Pharmacy, King Saud University, PO Box 2457, Riyadh 11451, Saudi Arabia; b Crystal Materials Research Unit, Department of Chemistry, Faculty of Science, Prince of Songkla University, Hat-Yai, Songkhla 90112, Thailand; c X-ray Crystallography Unit, School of Physics, Universiti Sains Malaysia, 11800 USM, Penang, Malaysia

## Abstract

In the title acrylonitrile derivative, C_17_H_14_N_2_OS, the central amino­acryl­aldehyde O=C—C=C—NH unit, wherein an intra­molecular N—H⋯O hydrogen bond generates an *S*(6) ring motif, is approximately planar, with an r.m.s. deviation of 0.0234 (2) Å for the five non-H atoms. This plane makes dihedral angles of 41.04 (9) and 84.86 (10)° with the two phenyl rings. The dihedral angle between the two phenyl rings is 54.82 (10)°. An intra­molecular C—H⋯N hydrogen bond is also present. In the crystal, weak C—H⋯π and π–π inter­actions, with a centroid–centroid distance of 3.8526 (14) Å, are observed.

## Related literature

For bond-length data, see: Allen *et al.* (1987 ▶). For hydrogen-bond motifs, see: Bernstein *et al.* (1995 ▶). For background to the synthesis and chemistry of acrylonitrile derivatives, see: Saufi & Ismail (2002 ▶); Sączewski *et al.* (2004 ▶); Sommen *et al.* (2002 ▶, 2003 ▶); Rudorf & Augustin (1977 ▶).
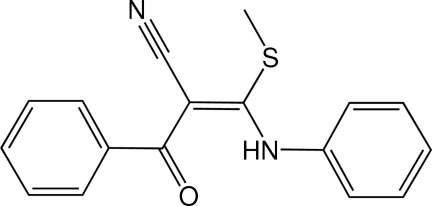


## Experimental

### 

#### Crystal data

C_17_H_14_N_2_OS
*M* *_r_* = 294.37
Monoclinic, 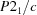

*a* = 8.7522 (2) Å
*b* = 10.8464 (3) Å
*c* = 16.1156 (4) Å
β = 103.968 (2)°
*V* = 1484.62 (7) Å^3^
*Z* = 4
Cu *K*α radiation
μ = 1.93 mm^−1^
*T* = 296 K
0.58 × 0.52 × 0.34 mm

#### Data collection

Bruker SMART APEXII CCD area-detector diffractometer
Absorption correction: multi-scan (*SADABS*; Bruker, 2009 ▶) *T*_min_ = 0.401, *T*_max_ = 0.556
9777 measured reflections
2603 independent reflections
2403 reflections with *I* > 2σ(*I*)
*R*_int_ = 0.024

#### Refinement

*R*[*F* ^2^ > 2σ(*F* ^2^)] = 0.039
*wR*(*F* ^2^) = 0.109
*S* = 1.04
2603 reflections
197 parameters
H atoms treated by a mixture of independent and constrained refinement
Δρ_max_ = 0.23 e Å^−3^
Δρ_min_ = −0.23 e Å^−3^

### 

Data collection: *APEX2* (Bruker, 2009 ▶); cell refinement: *SAINT* (Bruker, 2009 ▶); data reduction: *SAINT*; program(s) used to solve structure: *SHELXTL* (Sheldrick, 2008 ▶); program(s) used to refine structure: *SHELXTL*; molecular graphics: *SHELXTL*; software used to prepare material for publication: *SHELXTL* and *PLATON* (Spek, 2009 ▶).

## Supplementary Material

Crystal structure: contains datablock(s) global, I. DOI: 10.1107/S1600536812013475/is5105sup1.cif

Structure factors: contains datablock(s) I. DOI: 10.1107/S1600536812013475/is5105Isup2.hkl

Supplementary material file. DOI: 10.1107/S1600536812013475/is5105Isup3.cml

Additional supplementary materials:  crystallographic information; 3D view; checkCIF report

## Figures and Tables

**Table 1:** Hydrogen-bond geometry (Å, °) *Cg*1 is the centroid of the C1–C6 benzene ring.

*D*—H⋯*A*	*D*—H	H⋯*A*	*D*⋯*A*	*D*—H⋯*A*
N1—H1*N*1⋯O1	0.91 (3)	1.86 (3)	2.610 (2)	137 (2)
C11—H11*B*⋯N2	0.96	2.60	3.372 (2)	138
C17—H17*A*⋯*Cg*1^i^	0.93	2.91	3.690 (2)	143

Symmetry code: (i) 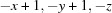.

**Fig. 1.**
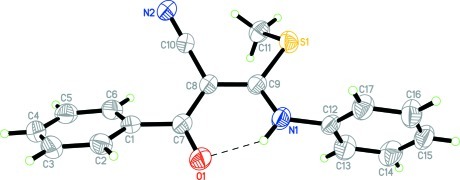
The structure of the title compound, showing 40% probability displacement ellipsoids and the atom-numbering scheme. The intramolecular N—H···O hydrogen bond is shown as a dash line.

**Fig. 2.**
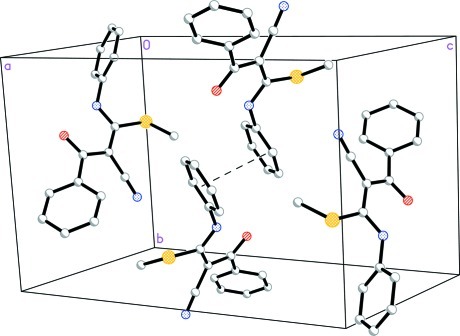
A crystal packing diagram of the title compound, viewed along the approximately along the a axis, showing the π–π interaction (dashed line) between the aminophenyl rings. H atoms were omitted for clarity.

## supplementary crystallographic information

### Comment

Acrylonitrile derivatives play many important roles in chemistry such as in membrane technology (Saufi & Ismail, 2002), synthesis and medicinal chemistry (Sączewski *et al.*, 2004; Sommen *et al.*, 2002, 2003). 2,3-Disubstituted acrylonitriles represent an interesting class of biologically active compounds, many of them possess cytotoxicity (Sączewski *et al.*, 2004). These interesting acrylonitrile derivatives promted us to synthesize the title acrylonitrile derivative (I) in order to study for its biological activity. Herein the crystal structure of (I) was reported.

The molecule of the title acrylonitrile derivative, C_17_H_14_N_2_OS, exists in an *E* configuration with respect to the olifinic C8═C9 double bond [1.412 (2) Å] and with torsion angles C10–C8–C9–N1 = 162.52 (16)° and C7–C8–C9–S1 = 173.43 (12)° (Fig. 1). The molecule is twisted with the dihedral angle between the two phenyl rings being 54.82 (10)°. Atoms of the middle fragment of the central aminoacrylaldehyde unit (C7–C9/O1/N1) lie roughly on the same plane with an r.m.s. deviation of 0.0234 (2) Å for the five non-H atoms (C7–C9/O1/N1). An intramolecular N1—H1N1···O1 hydrogen bond (Table 1) generates an S(6) ring motif (Fig. 1) (Bernstein *et al.*, 1995), which helps to stabilize the planarity of this plane. The mean plane through the C7/C8/C9/O1/N1 atoms makes dihedral angles of 41.04 (9) and 84.86 (10)° with the benzoyl and aminophenyl rings, respectively. The orientation of the methylthio group with respect to the olefinic moiety is indicated by the torsion angle C11–S1–C9–C8 = -42.56 (16)°. The dihedral angle between the two mean planes of N2/C10/C8/C9 and C8/C9/S1/C11 is 47.08 (15)°. An intramolecular weak C—H···N interaction generates an *S*(7) ring motif (Bernstein *et al.*, 1995). The bond distances of (I) are within normal ranges (Allen *et al.*, 1987).

The crystal packing of (I) is stabilized by weak C—H···π interactions (Table 1). A π–π interaction (Fig. 2) between the two aminophenyl rings with the distance of *Cg*2···*Cg*2^ii^ = 3.8526 (14) Å [symmetry code (ii) = 1-x, 1-y, -z] was presented; *Cg*2 is the centroid of the C12–C17 ring.

### Experimental

The title compound was prepared according to the reported method (Rudorf *et al.*, 1977). Single crystals of the title compound suitable for *X*-ray structure determination were recrystallized from ethanol by the slow evaporation of the solvent at room temperature after several days.

### Refinement

Amino H atom was located in a difference Fourier map and refined isotropically [N—H = 0.91 (3) Å]. The remaining H atoms were placed in calculated positions with d(C—H) = 0.93 for aromatic and 0.96 Å for CH_3_ atoms. The *U*_iso_ values were constrained to be 1.5*U*_eq_ of the carrier atom for methyl H atoms and 1.2*U*_eq_ for the remaining H atoms. A rotating group model was used for the methyl groups.

### Crystal data

**Table:**

C_17_H_14_N_2_OS	*F*(000) = 616
*M**_r_* = 294.37	*D*_x_ = 1.317 Mg m^−^^3^
Monoclinic, *P*2_1_/*c*	Cu *K*α radiation, λ = 1.54178 Å
Hall symbol: -P 2ybc	Cell parameters from 2603 reflections
*a* = 8.7522 (2) Å	θ = 5.0–67.5°
*b* = 10.8464 (3) Å	µ = 1.93 mm^−^^1^
*c* = 16.1156 (4) Å	*T* = 296 K
β = 103.968 (2)°	Block, colorless
*V* = 1484.62 (7) Å^3^	0.58 × 0.52 × 0.34 mm
*Z* = 4	

### Data collection

**Table:**

Bruker SMART APEXII CCD area-detector diffractometer	2603 independent reflections
Radiation source: fine-focus sealed tube	2403 reflections with *I* > 2σ(*I*)
Graphite monochromator	*R*_int_ = 0.024
φ and ω scans	θ_max_ = 67.5°, θ_min_ = 5.0°
Absorption correction: multi-scan (*SADABS*; Bruker, 2009)	*h* = −8→10
*T*_min_ = 0.401, *T*_max_ = 0.556	*k* = −12→12
9777 measured reflections	*l* = −19→19

### Refinement

**Table:**

Refinement on *F*^2^	Secondary atom site location: difference Fourier map
Least-squares matrix: full	Hydrogen site location: inferred from neighbouring sites
*R*[*F*^2^ > 2σ(*F*^2^)] = 0.039	H atoms treated by a mixture of independent and constrained refinement
*wR*(*F*^2^) = 0.109	*w* = 1/[σ^2^(*F*_o_^2^) + (0.0633*P*)^2^ + 0.3429*P*] where *P* = (*F*_o_^2^ + 2*F*_c_^2^)/3
*S* = 1.04	(Δ/σ)_max_ = 0.001
2603 reflections	Δρ_max_ = 0.23 e Å^−^^3^
197 parameters	Δρ_min_ = −0.23 e Å^−^^3^
0 restraints	Extinction correction: *SHELXTL* (Sheldrick, 2008), Fc^*^=kFc[1+0.001xFc^2^λ^3^/sin(2θ)]^-1/4^
Primary atom site location: structure-invariant direct methods	Extinction coefficient: 0.0100 (8)

### Special details

**Table:**

Geometry. All esds (except the esd in the dihedral angle between two l.s. planes) are estimated using the full covariance matrix. The cell esds are taken into account individually in the estimation of esds in distances, angles and torsion angles; correlations between esds in cell parameters are only used when they are defined by crystal symmetry. An approximate (isotropic) treatment of cell esds is used for estimating esds involving l.s. planes.
Refinement. Refinement of F^2^ against ALL reflections. The weighted R-factor wR and goodness of fit S are based on F^2^, conventional R-factors R are based on F, with F set to zero for negative F^2^. The threshold expression of F^2^ > 2sigma(F^2^) is used only for calculating R-factors(gt) etc. and is not relevant to the choice of reflections for refinement. R-factors based on F^2^ are statistically about twice as large as those based on F, and R- factors based on ALL data will be even larger.

### Fractional atomic coordinates and isotropic or equivalent isotropic displacement parameters (Å^2^)

**Table:**

	*x*	*y*	*z*	*U*_iso_*/*U*_eq_	
S1	0.26713 (5)	0.35557 (4)	0.12096 (3)	0.05369 (19)	
O1	0.76575 (17)	0.35293 (12)	0.07292 (10)	0.0674 (4)	
N1	0.48494 (19)	0.25937 (13)	0.05485 (10)	0.0525 (4)	
N2	0.4752 (2)	0.65992 (13)	0.16908 (13)	0.0639 (4)	
C1	0.84541 (18)	0.53504 (14)	0.14953 (10)	0.0427 (4)	
C2	0.9477 (2)	0.57125 (16)	0.10025 (11)	0.0520 (4)	
H2A	0.9370	0.5375	0.0461	0.062*	
C3	1.0652 (2)	0.65671 (18)	0.13054 (15)	0.0630 (5)	
H3A	1.1317	0.6813	0.0965	0.076*	
C4	1.0834 (2)	0.70502 (17)	0.21094 (15)	0.0649 (5)	
H4A	1.1628	0.7621	0.2316	0.078*	
C5	0.9841 (2)	0.66911 (18)	0.26115 (13)	0.0605 (5)	
H5A	0.9977	0.7016	0.3159	0.073*	
C6	0.8640 (2)	0.58504 (16)	0.23089 (11)	0.0507 (4)	
H6A	0.7964	0.5623	0.2648	0.061*	
C7	0.7250 (2)	0.43946 (15)	0.11312 (10)	0.0471 (4)	
C8	0.56634 (19)	0.45013 (14)	0.12432 (10)	0.0439 (4)	
C9	0.4545 (2)	0.35497 (13)	0.09959 (10)	0.0438 (4)	
C10	0.51581 (19)	0.56593 (14)	0.15039 (11)	0.0469 (4)	
C11	0.3008 (2)	0.40943 (17)	0.22941 (11)	0.0557 (4)	
H11A	0.2120	0.3887	0.2519	0.084*	
H11B	0.3146	0.4973	0.2307	0.084*	
H11C	0.3937	0.3711	0.2635	0.084*	
C12	0.3870 (2)	0.15146 (13)	0.03407 (11)	0.0446 (4)	
C13	0.4078 (3)	0.05512 (18)	0.09040 (12)	0.0640 (5)	
H13A	0.4838	0.0595	0.1418	0.077*	
C14	0.3149 (3)	−0.04908 (18)	0.07019 (14)	0.0696 (6)	
H14A	0.3281	−0.1148	0.1083	0.084*	
C15	0.2036 (3)	−0.05567 (17)	−0.00557 (13)	0.0624 (5)	
H15A	0.1411	−0.1256	−0.0189	0.075*	
C16	0.1846 (3)	0.04131 (19)	−0.06189 (13)	0.0680 (5)	
H16A	0.1087	0.0369	−0.1133	0.082*	
C17	0.2775 (3)	0.14547 (16)	−0.04263 (12)	0.0580 (5)	
H17A	0.2659	0.2106	−0.0812	0.070*	
H1N1	0.578 (3)	0.265 (2)	0.0390 (16)	0.083 (8)*	

### Atomic displacement parameters (Å^2^)

**Table:**

	*U*^11^	*U*^22^	*U*^33^	*U*^12^	*U*^13^	*U*^23^
S1	0.0462 (3)	0.0578 (3)	0.0579 (3)	−0.00653 (16)	0.01425 (19)	−0.01599 (17)
O1	0.0598 (8)	0.0556 (8)	0.0942 (10)	−0.0063 (6)	0.0328 (7)	−0.0289 (7)
N1	0.0539 (9)	0.0434 (7)	0.0649 (9)	−0.0093 (6)	0.0235 (7)	−0.0163 (6)
N2	0.0534 (9)	0.0381 (8)	0.1009 (13)	−0.0011 (6)	0.0200 (8)	−0.0108 (7)
C1	0.0400 (8)	0.0378 (7)	0.0502 (8)	0.0055 (6)	0.0105 (6)	0.0008 (6)
C2	0.0481 (9)	0.0536 (9)	0.0564 (9)	0.0045 (7)	0.0164 (7)	−0.0001 (7)
C3	0.0478 (10)	0.0568 (11)	0.0874 (14)	−0.0016 (8)	0.0224 (9)	0.0082 (9)
C4	0.0455 (9)	0.0486 (10)	0.0956 (14)	−0.0029 (7)	0.0071 (9)	−0.0058 (9)
C5	0.0538 (10)	0.0562 (10)	0.0638 (11)	0.0083 (8)	−0.0007 (8)	−0.0141 (8)
C6	0.0484 (9)	0.0517 (9)	0.0516 (9)	0.0048 (7)	0.0111 (7)	−0.0011 (7)
C7	0.0495 (9)	0.0400 (8)	0.0528 (9)	0.0006 (7)	0.0140 (7)	−0.0032 (6)
C8	0.0449 (8)	0.0358 (7)	0.0513 (8)	−0.0014 (6)	0.0123 (7)	−0.0048 (6)
C9	0.0474 (9)	0.0384 (8)	0.0448 (8)	0.0001 (6)	0.0094 (6)	−0.0016 (6)
C10	0.0422 (8)	0.0379 (8)	0.0602 (9)	−0.0042 (6)	0.0115 (7)	−0.0026 (7)
C11	0.0698 (11)	0.0484 (9)	0.0520 (9)	−0.0024 (8)	0.0207 (8)	−0.0035 (7)
C12	0.0482 (9)	0.0361 (8)	0.0520 (9)	−0.0015 (6)	0.0170 (7)	−0.0088 (6)
C13	0.0721 (12)	0.0561 (10)	0.0566 (10)	−0.0033 (9)	0.0014 (9)	0.0047 (8)
C14	0.0857 (14)	0.0452 (10)	0.0781 (13)	−0.0034 (9)	0.0200 (11)	0.0149 (9)
C15	0.0684 (12)	0.0427 (9)	0.0803 (13)	−0.0132 (8)	0.0260 (10)	−0.0120 (8)
C16	0.0713 (12)	0.0612 (11)	0.0638 (11)	−0.0144 (9)	0.0015 (9)	−0.0082 (9)
C17	0.0689 (12)	0.0443 (9)	0.0576 (10)	−0.0052 (8)	0.0089 (9)	0.0030 (7)

### Geometric parameters (Å, º)

**Table:**

S1—C9	1.7549 (17)	C6—H6A	0.9300
S1—C11	1.7983 (17)	C7—C8	1.447 (2)
O1—C7	1.241 (2)	C8—C9	1.412 (2)
N1—C9	1.326 (2)	C8—C10	1.428 (2)
N1—C12	1.441 (2)	C11—H11A	0.9600
N1—H1N1	0.91 (3)	C11—H11B	0.9600
N2—C10	1.144 (2)	C11—H11C	0.9600
C1—C2	1.389 (2)	C12—C13	1.367 (3)
C1—C6	1.392 (2)	C12—C17	1.371 (3)
C1—C7	1.494 (2)	C13—C14	1.385 (3)
C2—C3	1.383 (3)	C13—H13A	0.9300
C2—H2A	0.9300	C14—C15	1.367 (3)
C3—C4	1.371 (3)	C14—H14A	0.9300
C3—H3A	0.9300	C15—C16	1.373 (3)
C4—C5	1.379 (3)	C15—H15A	0.9300
C4—H4A	0.9300	C16—C17	1.383 (3)
C5—C6	1.388 (3)	C16—H16A	0.9300
C5—H5A	0.9300	C17—H17A	0.9300
			
C9—S1—C11	104.50 (9)	N1—C9—C8	120.54 (16)
C9—N1—C12	125.09 (15)	N1—C9—S1	115.44 (13)
C9—N1—H1N1	114.4 (17)	C8—C9—S1	123.98 (12)
C12—N1—H1N1	120.4 (17)	N2—C10—C8	178.1 (2)
C2—C1—C6	118.92 (15)	S1—C11—H11A	109.5
C2—C1—C7	117.55 (14)	S1—C11—H11B	109.5
C6—C1—C7	123.47 (15)	H11A—C11—H11B	109.5
C3—C2—C1	120.98 (17)	S1—C11—H11C	109.5
C3—C2—H2A	119.5	H11A—C11—H11C	109.5
C1—C2—H2A	119.5	H11B—C11—H11C	109.5
C4—C3—C2	119.76 (19)	C13—C12—C17	120.98 (16)
C4—C3—H3A	120.1	C13—C12—N1	119.33 (16)
C2—C3—H3A	120.1	C17—C12—N1	119.67 (15)
C3—C4—C5	120.06 (17)	C12—C13—C14	119.40 (18)
C3—C4—H4A	120.0	C12—C13—H13A	120.3
C5—C4—H4A	120.0	C14—C13—H13A	120.3
C4—C5—C6	120.69 (18)	C15—C14—C13	120.27 (18)
C4—C5—H5A	119.7	C15—C14—H14A	119.9
C6—C5—H5A	119.7	C13—C14—H14A	119.9
C5—C6—C1	119.58 (17)	C14—C15—C16	119.79 (17)
C5—C6—H6A	120.2	C14—C15—H15A	120.1
C1—C6—H6A	120.2	C16—C15—H15A	120.1
O1—C7—C8	122.07 (15)	C15—C16—C17	120.44 (18)
O1—C7—C1	117.75 (15)	C15—C16—H16A	119.8
C8—C7—C1	120.17 (13)	C17—C16—H16A	119.8
C9—C8—C10	118.88 (15)	C12—C17—C16	119.11 (17)
C9—C8—C7	121.80 (14)	C12—C17—H17A	120.4
C10—C8—C7	118.79 (14)	C16—C17—H17A	120.4
			
C6—C1—C2—C3	−0.8 (2)	C12—N1—C9—S1	−9.1 (2)
C7—C1—C2—C3	−178.16 (16)	C10—C8—C9—N1	162.52 (16)
C1—C2—C3—C4	1.2 (3)	C7—C8—C9—N1	−9.0 (2)
C2—C3—C4—C5	−0.5 (3)	C10—C8—C9—S1	−15.0 (2)
C3—C4—C5—C6	−0.7 (3)	C7—C8—C9—S1	173.43 (12)
C4—C5—C6—C1	1.0 (3)	C11—S1—C9—N1	139.77 (13)
C2—C1—C6—C5	−0.3 (2)	C11—S1—C9—C8	−42.56 (16)
C7—C1—C6—C5	176.88 (15)	C9—N1—C12—C13	−87.4 (2)
C2—C1—C7—O1	39.4 (2)	C9—N1—C12—C17	94.4 (2)
C6—C1—C7—O1	−137.81 (18)	C17—C12—C13—C14	−1.2 (3)
C2—C1—C7—C8	−139.89 (16)	N1—C12—C13—C14	−179.41 (19)
C6—C1—C7—C8	42.9 (2)	C12—C13—C14—C15	0.2 (3)
O1—C7—C8—C9	8.1 (3)	C13—C14—C15—C16	0.2 (3)
C1—C7—C8—C9	−172.59 (14)	C14—C15—C16—C17	0.2 (3)
O1—C7—C8—C10	−163.40 (17)	C13—C12—C17—C16	1.6 (3)
C1—C7—C8—C10	15.9 (2)	N1—C12—C17—C16	179.83 (18)
C12—N1—C9—C8	173.11 (16)	C15—C16—C17—C12	−1.1 (3)

### Hydrogen-bond geometry (Å, º)

*Cg*1 is the centroid of the C1–C6 benzene ring.

**Table:**

*D*—H···*A*	*D*—H	H···*A*	*D*···*A*	*D*—H···*A*
N1—H1*N*1···O1	0.91 (3)	1.86 (3)	2.610 (2)	137 (2)
C11—H11*B*···N2	0.96	2.60	3.372 (2)	138
C17—H17*A*···*Cg*1^i^	0.93	2.91	3.690 (2)	143

Symmetry code: (i) −*x*+1, −*y*+1, −*z*.
